# Signposts for School Refusal Interventions, Based on the Views of Stakeholders

**DOI:** 10.5334/cie.42

**Published:** 2022-05-18

**Authors:** David Heyne, Marije Brouwer-Borghuis

**Affiliations:** 1 Leiden University, NL; 2 SWV VO/VSO 2301 Almelo, NL

**Keywords:** School attendance, school refusal, intervention, mental health, education

## Abstract

School refusal (SR) signals a young person’s difficulty attending school. It jeopardizes their development, often contributes to distress for parents, and places an extra burden on school personnel. Reviews of empirical studies indicate that intervention for SR helps to increase school attendance, but not for all youths. This practice-based manuscript aims to support practitioners and organisations addressing the needs of youths and families affected by SR. Specifically, we present 14 signposts for the development and delivery of intervention for SR. The signposts represent important conditions for effective intervention based on key findings from the Knowing What Works project in the Netherlands. During that project, 76 professionals shared their views about the important elements in SR interventions they delivered, and 39 youths and 86 parents shared their views about the helpful elements in SR interventions in which they participated. These 201 stakeholders were variously associated with 21 SR interventions across 9 of the 12 Dutch provinces, most situated in mainstream or special education settings. Their responses informed the development of the 14 signposts presented here, supported by the extant literature on SR intervention. We describe the essence of each signpost and conclude with suggestions for using the signposts and evaluating their utility.

## The Advantages of School Attendance

Being at school offers youths immediate positive experiences such as time with friends, learning something interesting, and feeling cared for by a teacher. The short- and long-term benefits of school attendance are similarly diverse. School is a context in which youths can use and develop social and emotional competencies such as skills related to relationships, decision-making, and self-regulation (Collie, 2020). Regarding self-regulation, school attendance fosters routines and responsibilities such as getting up in the morning to arrive at school on time and observing the norms for behaviour during school time (Heyne, Gentle-Genitty, et al., 2020). Exposure to instructional time benefits intellectual development, academic achievement, and educational outcomes (Allensworth & Balfanz, 2019; Ginsburg et al., 2014; Keppens & Spruyt, 2020). Positive health outcomes are allegedly influenced by the roles youths can have at school (Bonell et al., 2019) and by the social, emotional, and academic functioning they develop during the school years (Okano et al., 2019; Panayiotou et al., 2021). Depending on the curriculum, school attendance can influence identity, passions, morals, and ethics (Eccles & Roeser, 2011). Youths more likely to graduate from school are those with better attendance (Schoeneberger, 2012; Smerillo et al., 2018) and better school performance (Allison et al., 2019), the latter influenced by the former. The benefits of graduation include preparation for successful transition to adulthood (Fredricks et al., 2019), such as social and economic participation in society (Zaff et al., 2017).

## School Refusal and Its Impact

School refusal (SR) refers to a young person’s reluctance or refusal to attend school, in combination with emotional distress (Heyne et al., 2019). The young person does not hide their absence from parents, a criterion that helps differentiate SR from truancy. Parents have made efforts to secure school attendance or express intentions for their child to attend school, a criterion that helps differentiate SR from school withdrawal. School personnel are eager to support the young person’s attendance at school, which is different to school exclusion. In short, SR is differentiated from these other types of attendance problems because the different types are held to be influenced by different variables and in need of different interventions (Heyne et al., 2019).

School refusal occurs among 1–7% of youths in the general population, varying according to the definition used and sample studied. Heyne and King’s (2004) narrative synthesis indicated a prevalence rate of 1–2% across all school-aged youths. The Egger et al. (2003) community study of 9- to 16-year-olds yielded a similar rate across a three-month period. Havik et al. (2015) found that 3.6% of 11- to 15-year-olds reported absence from school in the last three months that was quite often because of SR-related reasons. The Steinhausen et al. (2008) community sample of 11- to 17-year-olds revealed that 6.9% reported fear of going to school. Rates of SR among youths seen in clinical settings range from 5–16% (Al Husni Al Keilani & Delvenne, 2021; Burke & Silverman, 1987; Hersov, 1985; Honjo et al., 1992; McShane et al., 2001; Roué et al., 2021).

School refusal has both short- and long-term negative effects for youths, families, schools, and the community. Many of the negative effects for youths stem from the absenteeism associated with SR, which is often weeks, months, or years. Indeed, most youths participating in the 21 interventions reported by Heyne, Brouwer-Borghuis, et al. (2022) were absent between three months and one year prior to intervention. Absence is associated with lowered academic achievement (e.g., Klein et al., 2021) and predicts school dropout (Schoeneberger, 2012), which is associated with unemployment (Attwood & Croll, 2006) and lower life expectancy (Rogers et al., 2013). In addition, there are indications that absenteeism can impair youths’ social-emotional development (Gottfried, 2014).

Parents of youths displaying SR experience frustration and helplessness, confronted with the dilemma of finding “the right balance between how much the child is capable of and how much the parents should challenge the child” (Dannow et al., 2020, p. 31). The family is under great stress because the young person’s difficulty attending school usually begins in the home environment, such as complaints about school and symptoms of anxiety (Berry & Lizardi, 1985). The practical impact of SR accompanies the emotional impact, such as spending time communicating with the school (e.g., writing absence notes, asking for help) and addressing other consequences such as planning private lessons and arranging visits to the doctor (Gallé-Tessonneau & Heyne, 2020). In addition, conflict can occur between parents with different ideas about how to respond to a child’s refusal to attend, and between the young person and their parents (Heyne, 2021).

Education professionals report that the management of attendance problems is resource-intensive and emotionally challenging (Finning et al., 2018). Time is needed to help youths catch up on missed learning and to plan, implement, and evaluate strategies to support absent youths. Professionals external to the school may also be involved. For example, community-based family coaches may help school personnel adapt the school environment to help youths feel safe when back at school (Tobias, 2019). For society, lower rates of school completion contribute to reduced productivity and increased social support costs (Evans, 2000).

Youths displaying SR may have a diagnosable mental health problem, but this is not inherent to SR. For example, some youths experience clinical levels of separation anxiety, social anxiety, or depression (Heyne et al., 2002) while others do not meet criteria for any mental health problem (Egger et al., 2003). A broad range of influences at the individual, family, school, and community levels can predispose youths to the development of SR, precipitate its onset, perpetuate the problem, and serve as protective factors (Heyne et al., 2014). SR is thus used to describe the phenomenon characterised by a youth’s difficulty going to school, not to ascribe causation to the young person. Following Devenney and O’Toole (2021), it is argued that the responsibility for addressing SR does not lie at the feet of the young person or family. Rather, professionals work together with youths, families, schools, and support services to address the broad range of influences and subsequent interventions.

## Interventions for Severe or Chronic School Refusal

In the field of school attendance, interventions are increasingly organised according to three tiers in the multidimensional, multi-tiered system of supports model (Kearney & Graczyk, 2020). Tier 1 interventions aim to promote attendance and prevent absenteeism; Tier 2 interventions focus on efficient responding when attendance problems are emerging, mild, or moderate; and Tier 3 interventions involve intensive support when attendance problems are severe or chronic. The current paper addresses severe or chronic SR (i.e., Tier 3 SR).

### Interventions Delivered by Mental Health Professionals

Many SR interventions are psychological in nature and delivered in clinical settings (Johnsen et al., 2021). For example, seven of the eight interventions in the review by Maynard et al. (2018) were variants of cognitive-behavioural therapy (CBT), and five of the eight were conducted in clinical settings. The tradition of understanding and responding to SR as a predominantly psychological problem likely has its roots in the tendency to characterise SR by the presence of emotional distress around attendance. The preponderance of interventions in clinical settings may be explained by the tradition of not providing mental health interventions in school settings, until quite recently (Heyne, Kearney, et al., 2022).

A summary of SR interventions was presented in a review by Heyne, Strömbeck, et al. (2020). It is unclear how many youths in the 51 studies reviewed displayed Tier 3 SR rather than Tier 2 SR because study authors did not employ this distinction, which is understandable because the model was only introduced in the last decade (Kearney & Graczyk, 2014). However, descriptions of the youths included in the studies indicate that some, and perhaps many, exhibited severe or chronic SR. For example, the average duration of SR was 56 weeks in the study by Last et al. (1998); the average amount of absence among youths was 85% in Melvin et al. (2017); and 64% of youths in Heyne et al. (2002) had experienced multiple episodes of SR.

The most common intervention in the review by Heyne, Strömbeck, et al. (2020) was a variant of CBT (44%), which often targets youth anxiety and depression and builds skills and confidence for re-engaging with school (Heyne, 2022). By helping youths manage emotional distress and increase school attendance, the broader aim of intervention for SR can be achieved: social-emotional and academic development (Heyne & Sauter, 2013).

CBT with youths is accompanied by close work with the family and school personnel (Elliott & Place, 2019). For example, CBT manuals for SR include family work on communication and problem solving (Heyne & Rollings, 2002; Heyne & Sauter, 2013; Kearney & Albano, 2018; Tolin et al., 2009). These manuals also emphasise work with parents such as the establishment of regular household routines, minimisation of home-based reinforcement during school hours, effective instruction giving, and increased confidence in managing SR. The manuals encourage practitioners to address school influences on the development, maintenance, and remediation of SR by working with school personnel. Topics include school-based modifications to accommodate the young person academically, socially, and emotionally (e.g., Heyne & Rollings, 2002); positive reinforcement in the school setting to promote attendance (e.g., Last, 1993); daily communication between teachers and parents (e.g., Kearney & Albano, 2018); helping school personnel consider the ways therapeutic gains can be maintained in the school setting (e.g., Tolin et al., 2009); and increasing school personnel’s understanding and motivation by reviewing the case formulation with them (e.g., Heyne & Sauter, 2013).

Interventions other than CBT were also identified in the review by Heyne, Strömbeck, et al. (2020). Twenty-five percent of studies evaluated some form of psychosocial intervention in addition to or instead of CBT, such as narrative therapy, motivational interviewing, multimodal treatment, parent counselling, collage therapy, and hypnosis. Sixteen percent evaluated medication as a stand-alone intervention, combined with CBT, or combined with other interventions such as individual psychotherapy for youths and casework with parents.

There are also numerous reports of family therapy for SR (e.g., Bryce & Baird, 1986; Richardson, 2016). It is not clear if mental health professionals conducted the family therapy, but the settings in which the intervention was delivered were mental health settings (i.e., a child and family psychiatric service; youth mental health service).

### Interventions Delivered by Education Professionals

There are few published examples of SR intervention delivered predominantly or exclusively by education professionals in school settings or other educational services. An exception is Preece and Howley’s (2018) alternative educational program for adolescents with autism spectrum disorder and anxiety-based absenteeism. Located in a small school building, the program was part of a service for students not attending mainstream school due to complex medical or mental health conditions, staffed by a class teacher, and supplemented with specialist subject teachers as appropriate. Youths were helped to prepare for re-engagement with formal education via attention to their wellbeing, relationship building, frequent face-to-face communication between staff and the family, and autism-friendly surroundings (e.g., structured classroom layout and clarity about the functions of the different areas). There was minimal daily input from external mental health professionals (i.e., parent group training in anxiety management). However, professionals in the program used different theoretical approaches that are considered “good practice” when working with young people with autism. In addition, there was collaboration between team members, external professionals and parents. Two other examples of SR intervention delivered by education professionals include an alternative school for youths with school-related anxiety (Wilkins, 2008) and behaviour modification applied by an elementary school principal (Brown et al., 1974).

Interventions conducted in school settings by education professionals are well suited to addressing school influences on the development, maintenance, and remediation of SR. Of particular note are the influences found in Ingul et al. (2019), which includes a review of the characteristics of school settings that have been associated with SR. These include the classroom situation (e.g., problematic student-teacher relationship; lack of teacher support; fear of the teacher; noisy and unpredictable classrooms); general aspects of the school (e.g., fear of less structured aspects such as break times; the sense of attending a dangerous school); social aspects (e.g., bullying, difficulty making friends, feeling isolated); and educational aspects (e.g., anxiety about academic performance and a mismatch between the young person’s ability and the academic demands of school).

### Interventions Delivered by Mental Health and Education Professionals

The literature also includes examples of multidisciplinary efforts to meet the needs of youths with severe or chronic SR. McShane et al. (2007) reported on the Sulman Program in Australia, an adolescent mental health and education program located at a special education school adjoining a psychiatric unit. The program was designed to better meet the needs of socially anxious adolescents with anxiety-based school attendance problems. Walter et al. (2010) reported on an inpatient mental health service in Germany, which included access to a school for special education. The intervention was designed to provide support to adolescents with chronic anxious-depressive absenteeism, including parent- and teacher-focused interventions. In severe cases (i.e., complete absence for more than three months), intervention included attendance at the special school. Grandison (2011) reported on a Short Stay School program in England for youths with medical and mental health needs, most of whom were adolescents displaying SR. The program was staffed by professionals from education and health services and jointly funded by both services.

More recently, three interventions were described in a special issue of *Cognitive and Behavioral Practice*. The Multimodal Treatment in Germany was developed for youths referred to a mental health setting who display SR, truancy, or both (Reissner et al., 2019). The program was developed by a multidisciplinary team (i.e., psychotherapists, psychiatrists, psychiatric nurses, social workers, teachers, and a sports scientist), which also delivers the intervention and engages in regular case conferences. Further, the Link in the Netherlands is an alternative educational program for adolescents displaying SR (Brouwer-Borghuis et al., 2019). The program is based on close collaboration between professionals from education and specialist youth care. For example, therapists from a mental health service visit the young people in the Link class. Funding comes from education, the municipality, and a national government initiative to reduce early school leaving. Finally, the In2School intervention in Australia was also designed for youths displaying SR (McKay-Brown et al., 2019). It is housed in a special education facility located at a mental health service and provided in kind by that service. Researchers, teachers, and mental health clinicians developed the intervention together because of the range of problems youths displaying SR can experience. The education- and health-focused partnership is underscored by the daily collaboration between the teachers and a clinician, the latter role variously filled by a social worker, psychiatric nurse, or psychologist.

## Evaluations of Interventions for Severe or Chronic School Refusal

Empirical studies advance knowledge about the effectiveness of SR interventions via robust scientific evaluation. Researchers have conducted reviews of these studies. King et al. (2005) presented a narrative review of CBT for SR based on seven studies. They concluded that CBT appears to be useful. Silverman et al. (2008) also presented a narrative review of psychosocial interventions for SR, using stricter criteria for study inclusion. On the basis of four studies, they concluded that child/adolescent-focused CBT, parent-focused CBT, and their combination, are possibly efficacious. It appears this conclusion was based on youth and parent reports of anxiety and other symptoms (e.g., depression) and not on school attendance data. Pina et al. (2009) presented a narrative review of eight single-case experimental design studies and six group-design studies of psychosocial interventions for SR. They reported significant increases in attendance and reductions in symptoms associated with SR (e.g., anxiety, depression, disruptive behaviour) and suggested that behavioural strategies alone or in combination with cognitive strategies are promising lines of intervention. Their review included an evaluation of effect sizes based on five group-design studies, yielding two conclusions: (a) interventions yield significant increases in attendance and reductions in symptoms, but there is room for improving the efficacy of interventions; and (b) effects can be achieved by working directly with the young person, working directly with parents and teachers, or both.

Maynard et al. (2018) conducted the most robust evaluation of psychosocial interventions for SR to date, only including studies with (a) a pre-post design in the context of a randomised controlled trial or quasi-experimental design; and (b) baseline data on outcomes or statistical controls. Different from the earlier reviews, the researchers included unpublished dissertations to provide a more comprehensive view of available evidence. Eight studies were included, seven of which evaluated CBT. The eighth study addressed non-directive Rogerian group-based counselling in a school setting. Across the eight studies there was a robust positive and significant effect for school attendance, but no significant effect for anxiety. The authors concluded that there is tentative support for CBT as an intervention for SR, noting that increased attendance could lead to an increase in anxiety, at least in the short-term (i.e., at posttreatment). They called for studies with long-term follow-up to determine whether increased attendance is accompanied, in the longer term, by decreased anxiety.

Collectively, four reviews indicated that psychosocial interventions contribute to positive outcomes, and CBT is the most commonly evaluated intervention. Despite the promise of CBT, a sizable group of youths is not helped by current interventions, and there is still a great need to improve intervention (Heyne, 2019). Outcomes for adolescents displaying SR appear to be inferior to those for children, with non-response to CBT and other interventions ranging from one third to two thirds of adolescents (Heyne, 2021).

After decades of research on SR interventions, there is still a need to increase knowledge, including better understanding of what contributes to change when SR interventions are employed (Maynard et al., 2018; Pina et al., 2009). Empirical studies predominantly focus on outcomes using quantitative methods (Heyne, Strömbeck, et al., 2020), evaluating interventions delivered in research settings to participants fulfilling stringent criteria for inclusion (Johnsen et al., 2021) and paying little attention to why interventions yield these outcomes (Heyne et al., 2015). By contrast, qualitative studies based on stakeholders’ views of intervention help shed light on what it is about interventions that make them work, thereby enriching an understanding of the complexities of interventions delivered in real-world settings (e.g., schools, clinics). According to the International Network for School Attendance, “contemporary models for understanding and reducing absenteeism will be enhanced by the voices of all stakeholders,” including youths, parents, and professionals (Heyne, Gentle-Genitty et al., 2020, p. 1026).

## Aim of the Current Paper

This practice-based manuscript is designed to support practitioners and organisations addressing the needs of youths and families affected by SR. Specifically, we present 14 signposts for the development and delivery of intervention for SR. The signposts represent important conditions for effective intervention based on qualitative data gathered during the Knowing What Works project in the Netherlands.

## Signposts for Developing and Delivering Interventions for School Refusal

### The Knowing What Works Project

The signposts described in the next section emerged from the Knowing What Works project (Heyne, Brouwer-Borghuis, et al., 2022). The project addressed the question of “what works” in intervention for SR, as a response to the need for a more coordinated approach to intervention according to the Dutch Expertise Network for School Attendance. Research activities associated with the Knowing What Works project were approved by the Psychology Ethics Committee of the Institute of Psychology at Leiden University.

In short, the project employed a mixed-methods design, combining the advantages of qualitative and quantitative methodologies. Seventy-six professionals delivering interventions were asked about which elements in intervention they regarded as important. Professionals included (school) psychologists (33%), teachers (29%), counsellors and coaches (13%), managers (9%), support staff (9%), education consultants (4%), and unspecified (3%). In addition, 39 youths and 86 parents participating in interventions were asked about the elements in intervention they regarded as helpful. Collectively, these 201 stakeholders were associated with 21 SR interventions delivered in 9 of the 12 Dutch provinces. Nineteen of the 21 interventions were situated in mainstream or special education settings, sometimes involving close collaboration with internal or external support services (e.g., youth care, mental health). Two interventions were situated in support services.

The qualitative data were gathered via 21 group interviews with professionals and via questionnaires developed for youths and parents. These qualitative data were thematically analysed following Braun and Clarke’s (2006) method. Emerging themes were organised in separate networks based on professionals’, youths’, or parents’ responses, and these themes were used to develop the signposts for school refusal interventions.

In addition, quantitative data were gathered via the questionnaires developed for youths and parents to assess the impact of the interventions. Two thirds to three quarters of youths and parents reported positive changes attributed to the intervention (e.g., reduced anxiety, stress, mood problems, difficulty attending school; increased school attendance, fun at school; improved problem solving; increased life satisfaction, confidence in the future), and two thirds of parents reported less stress regarding their child’s school attendance and greater capacity to support their child’s attendance. These retrospective reports of youths and parents do not constitute robust scientific support for the effectiveness of the interventions, but they do suggest that the interventions included in the Knowing What Works project have a positive effect.

### The Signposts for School Refusal Interventions

The signposts for SR interventions represent a synthesis of emerging best practices according to professionals, youths, and parents who participated in the Knowing What Works project, and emerging best practices identified in prior research. Preparation of the set of signposts is detailed in Heyne, Brouwer-Borghuis et al. (2022). In short, four members of the project consortium reviewed the literature and results in the Knowing What Works report. The four consortium members independently nominated 10 signposts. Of the 15 signposts identified across the four members, 11 were nominated by at least two members and 4 by a single member. Consensus meetings yielded a final set of 14 signposts (the 15th signpost was related to prevention and early intervention and fell outside the final set of signposts focused on intervention for Tier 3 SR). The signposts are presented in Table 1.

**Table 1:** Signposts for School Refusal Interventions.


1	Provide an integrated approach, including the young person, parents, and school

2	Pursue insight into the integrative picture

3	Invest in your availability and the quality of contact with the young person and parents

4	Promote the willingness and involvement of the young person and parents

5	Create a safe environment

6	Lower the hurdles in the beginning

7	Provide rhythm and structure

8	Broaden educational options and adjust educational tasks

9	Facilitate social contact with peers

10	Create movement

11	Work together as education and support services

12	Specify the method

13	Gather a committed team of professionals with knowledge and experience

14	Provide sufficient resources to implement the intervention


The signposts are applicable for severe and chronic SR (i.e., Tier 3 SR). The reader is reminded of the importance of interventions to promote attendance and prevent SR (Tier 1) and to respond efficiently when SR is emerging, mild, or moderate (Tier 2), discussed in the Knowing What Works report (Heyne, Brouwer-Borghuis, et al., 2022). The signposts for Tier 3 SR are not intended as a prescription for how to deliver intervention on a case-by-case basis nor which strategies or techniques should be used in relation to each signpost (e.g., CBT or eye movement desensitization and reprocessing). Rather, the 14 signposts signal important conditions for the development and delivery of effective intervention. Signposts 1 to 10 are directly relevant for professionals delivering intervention, Signposts 11 and 12 require the attention of professionals delivering intervention and management teams, and Signposts 13 and 14 are directly relevant for management teams.

The essence of each signpost is described next. To learn about the justification for signposts, links between them, and tips for working with them, the reader is referred to Heyne, Brouwer-Borghuis, et al. (2022).

#### Signpost 1 – Provide an Integrated Approach, Including the Young Person, Parents, and School

Signpost 1 signals the need for comprehensive and integrated intervention. Professionals spend time with the young person and parents, give attention to school-related matters, and ensure coherence between the work conducted with the young person, parents, and school personnel. Their individual work with the young person and parents is accompanied by their work with the young person and parents together, especially to address family communication and problem solving. As needed, professionals arrange extra support for parents to address parent-related problems and broader issues for the family (e.g., limited social network). Professionals also closely support school personnel in their role in intervention, be they personnel from the original school, a new school identified for the young person, or an alternative educational program serving as the SR intervention. Professionals delivering intervention in clinical settings provide consultation to school personnel while professionals in an educational intervention for SR conduct school-related interventions themselves. In an integrated approach, professionals also pay attention to the working relationship between school personnel, on the one hand, and the young person and parents, on the other hand. By specifying the frequency and nature of interactions between the family and school personnel, professionals safeguard attention to the working relationship between them.

#### Signpost 2 – Pursue Insight Into the Integrative Picture

Prior to starting intervention, professionals take time to understand which of the factors associated with absenteeism and SR are contributing to difficulties for the young person and family, and which difficulties are encountered by school personnel. Thus, professionals engage in discussion with youths, parents, school personnel, and others who know the young person and the family situation (e.g., current and prior helping professionals). When professionals understand the young person and their social context, they are in a better position to tailor intervention to the needs of the young person, family, and school. For example, how does a youth’s autism influence the manner in which intervention is conducted with them, what are the parents’ concerns and expectations regarding their autistic child’s education, and which educational environment is most suitable? The integrative picture is expanded during the course of intervention, as more information becomes available and the situation changes.

#### Signpost 3 – Invest in Your Availability and the Quality of Contact With the Young Person and Parents

The quality of contact with youths and parents warrants as much attention as the specific interventions employed. Relationship-based contact relies upon professionals’ availability and is characterised by a heartfelt commitment to those affected by SR, interest in the person as a whole, positive attention, nurturance and emotional support, empathy, acceptance, trustworthiness, patience and persistence, and inclusion in decision-making. This helps participants feel safe, supported, and taken seriously. It builds a sense of belonging and hope and enables professionals to foster helpful attitudes towards intervention (i.e., Signpost 4).

Attention to relational aspects is important from the outset (when distressed and discouraged youths and parents are most in need of understanding, support, and hope) and continues throughout intervention as participants develop trust in professionals. In educational interventions for SR, smaller group size (e.g., 10 youths per class) increases professionals’ availability and the quality of contact with each participant. Further, outreach (e.g., home visits) can enhance the quality of contact. Professionals also focus on the quality of contact with school personnel and other professionals involved in intervention, given the impact that a young person’s absenteeism can have upon them and the importance of their participation in intervention.

#### Signpost 4 – Promote the Willingness and Involvement of the Young Person and Parents

Signpost 4 underscores the importance of promoting youths’ and parents’ engagement with intervention, including initial willingness to participate and continued active involvement throughout. At the outset, professionals ascertain each participant’s level of engagement and readiness for change. This attention to engagement continues during intervention, to promote participation in the successively challenging steps associated with increasing school attendance. Attention to engagement is especially important when participants harbour unhelpful attitudes towards problems and school attendance, perhaps due to the experience of chronic SR, minimal prior support or progress, and thus frustration and hopelessness.

The quality of contact (i.e., Signpost 3) promotes engagement. Psychoeducation supports engagement by helping participants appreciate the relevance and importance of intervention and its components. Additional interventions may be needed such as motivational interviewing, home visits, arranging transport to the intervention, and technologies (e.g., virtual reality). Professionals also consider ways in which external professionals such as teachers and mentors can best be supported in their roles to shore up their willingness and involvement during intervention.

#### Signpost 5 – Create a Safe Environment

Professionals provide an intervention environment in which participants feel safe. Youths and parents who participate in SR intervention are likely to have experienced considerable discomfort, distress, and sometimes trauma as a result of SR or associated experiences (e.g., bullying for youths; embarrassment and confusion for parents).

Interventions in the form of alternative educational programs represent a key opportunity to provide youths with a safe environment in which to re-engage with schooling. During, after, or instead of participation in an alternative educational program, many youths are helped to re-engage with a mainstream school setting. This signals the need for professionals to consult with the school personnel in that setting about ways to help youths feel safe. When SR intervention is delivered in a clinical setting, professionals also pay attention to ways in which youths and parents are helped to feel safe. Results from the Knowing What Works project and the supporting literature indicate that professionals promote safety via attention to the physical setting (e.g., small group size increases tranquillity and safety) and the professional’s approach when meeting with youths and parents (e.g., acceptance, trustworthiness).

#### Signpost 6 – Lower the Hurdles in the Beginning

Signpost 6 signals the need for professionals to lower expectations for a rapid return to school when SR is severe or chronic. Initially, time is spent building relationships: professionals with youths and parents; youths and parents with each other and school personnel; and youths with peers. When professionals understand which factors “push” a young person away from school and “pull” them towards school, appropriate adjustments can be made. Professionals also use this time to help youths and parents develop skills for subsequently increasing and coping with attendance at school. It is important to note that lowering the hurdles in the beginning is not an end in itself, but it lays the groundwork for creating movement (Signpost 10). For example, lowering the hurdles benefits the quality of contact, participant engagement, and a sense of safety, thereby providing the context for professionals to work with youths and parents on creating movement.

Lowering the hurdles is applicable to all new experiences, not just the experience of increasing school attendance. For example, professionals lower expectations when youths commence intervention, first join in a group activity, and first re-engage with academic tasks.

#### Signpost 7 – Provide Rhythm and Structure

“Rhythm” here refers to participants’ experience of routines and predictability whereas “structure” refers to the arrangements professionals make to promote rhythm. These two elements are important for three main reasons. First, when SR is severe or chronic, youths’ and families’ routines have often become disrupted (e.g., sleeping in on school mornings, excessive gaming during the day, parents arriving late to work) and youths’ engagement in educational activities is disrupted. These disruptions maintain SR: Youths find it harder to attend school, and parents find it harder to manage SR. Second, many youths with SR experience anxiety, depression, autism, or a combination. Rhythm and structure provide extra predictability and security, helping youths engage in intervention and develop confidence and competencies. Third, rhythm and structure are inherent in the process of incrementally increasing school attendance.

At a macro level, predictability and security are increased when interventions have a broad structure (e.g., time for the young person to feel comfortable in the intervention, then developing coping skills, then engaging in more demanding activities such as increasing school attendance). The structure associated with daily participation in an alternative educational program similarly provides a sense of rhythm. At a micro level, rhythm is achieved via daily routines in both the intervention setting (e.g., morning check-in with the mentor) and the home environment (e.g., improved evening and morning routines). Predictability and security are also enhanced via clarity (e.g., professionals help participants understand the process associated with a gradual increase in school attendance) and via professionals’ reliability. In the words of a professional in the Knowing What Works project, “You say what you do and you do what you say, every day.”

#### Signpost 8 – Broaden Educational Options and Adjust Educational Tasks

Developing individual educational pathways for youths requires a thorough understanding of their capacities and potential and the extent to which their difficulty going to school is related to educational requirements. Professionals identify and implement necessary changes to the young person’s education. This may involve a broadening of educational options (e.g., referral to an alternative educational program; a curriculum better suited to the young person) or adjustments to the current educational program (e.g., reduced expectations for assignments or testing). Changes may be short or long term, enacted in the interest of the youth’s prospects for continued education.

If a young person attends an alternative educational program, academic tasks and progress are discussed with personnel from the original school and the school to which the young person will transition after the alternative program.

#### Signpost 9 – Facilitate Social Contact With Peers

Social problems may contribute to the development of SR, co-occur with SR, and arise out of absence from school. While the title of this signpost is simple, the process can be quite complex. Usually, it is not just a matter of creating opportunities for youths to spend time with other youths. Rather, youths typically require substantial support before, during, and following an increase in social contact. Issues to address might include social anxiety, low motivation for social interaction (e.g., when youths are depressed), and the acquisition of micro-skills (e.g., eye contact, tone of voice), macro-skills (e.g., initiating conversations, requesting help), and social-cognitive skills (e.g., interpreting social cues, interpersonal problem solving). Professionals support school personnel and parents as they support the young person’s social involvement inside and outside school.

Data from the Knowing What Works project and other qualitative studies point to the value of increasing social contact in a gradual fashion, and the added value of contact with peers with similar difficulties. The relationship between SR and bullying indicates a need for school-wide intervention (e.g., preventing and responding to bullying) alongside youth-focused intervention (e.g., social skills related to assertiveness).

#### Signpost 10 – Create Movement

Signpost 10 signals the need for professionals to create movement towards re-engagement with school. It is about breaking entrenched patterns of avoidance due to anxiety or inactivation due to depression. Re-engagement with the school setting is usually a gradual process, especially for Tier 3 SR. There are many possible gradations of re-engagement, such as increased time at school, increased time first in favoured classes and then less favoured classes, and increased expectations regarding schoolwork. Gradual re-engagement with school is often preceded by gradual change in other parts of the young person’s life, such as increased social involvement.

Practice tasks create movement by providing opportunities to apply the knowledge and skills youths learn during intervention to real-world settings, thus effecting change outside the intervention setting. Professionals also create movement by building a youth’s perspective for the future, helping parents acquire and use strategies that support re-engagement with school (e.g., establishing healthy morning and evening routines), and helping school personnel (e.g., creating a safe environment at school; adjusting educational tasks).

#### Signpost 11 – Work Together as Education and Support Services

Professionals from education and support services (e.g., mental health) need to establish effective and efficient collaboration to best meet the multiple needs of youths displaying SR and their families. “Collaboration between professionals” refers to the working relationship between two or more professionals. “Collaboration between organisations,” in turn, refers to arrangements between sectors or services involved in delivering intervention. Collaboration may be incidental, such as when professionals come together to address the needs of a specific young person and their family. Ideally, however, collaboration has a permanent structure, implying a verbal or written agreement between organisations ensuring that collaborating parties can rely upon each other on an ongoing basis. Whether collaboration is incidental or permanent, it is imperative that there be clear and regular communication about goals, roles, and progress. In this way, professionals ensure optimal support for youths and families.

#### Signpost 12 – Specify the Method

Signpost 12 encourages professionals to document their intervention. This includes a statement of the overarching goal(s) of intervention, specific objectives, and the methods used, together with hypothesised links between methods, objectives, and goal(s). Results of the Knowing What Works project suggest that flexibility and standardisation are both important in intervention for SR. Specifying the method sheds light on why, when, and how professionals employ flexibility in their work vis-à-vis working in a more standardised way, likely enhancing the implementation of an intervention. The contemporary emphasis on personalised intervention for each young person and family does not mean that professionals need to start from scratch in planning intervention for a specific young person and family. Rather, a framework in the form of goals, objectives, and methods provides direction in the process of personalizing intervention. It also supports the training of new team members and roll-out of the intervention.

#### Signpost 13 – Gather a Committed Team of Professionals With Knowledge and Experience

There are many influences on SR, and severe or chronic SR can be difficult to address. Teams providing intervention should comprise members with rich and diverse expertise based on knowledge and experience in addressing SR and working with youths, parents, and school personnel. The ideal characteristics of team members are those that support the work associated with other signposts, such as developing a holistic understanding (Signpost 2), developing quality contact with youths and parents (Signpost 3), helping youths and parents feel safe (Signpost 5), facilitating youths’ social contact (Signpost 9), and facilitating an increase in school attendance (Signpost 10). Other characteristics that emerged from the Knowing What Works project include vision, creativity, and openness to engage in professional development and quality control. Finally, teamwork is also important because intervention calls for sophisticated understanding, commitment to multiple disciplinary work, and moral support.

#### Signpost 14 – Provide Sufficient Resources to Implement the Intervention

Signpost 14 requires the attention of the management team responsible for providing sufficient resources to develop and deliver an intervention that adequately addresses the multiple needs of youths and families. Funding supports the provision of physical resources, human resources, and time. Physical resources might include a well-equipped classroom or other location for intervention, options for relaxation (e.g., a table-tennis table), and a comfortable space for parents to meet. The physical proximity of education and mental health services was also valued by professionals in the Knowing What Works project. Human resources include professionals with expertise related to SR, a sufficient number of professionals so intensive support can be offered, and professional development opportunities. Time is needed to develop the intervention (e.g., components of an alternative educational program), document it, deliver it (e.g., sufficient time with youths and parents, time to collaborate with external professionals, intervention long enough to yield enduring change), evaluate outcomes, and share accumulated knowledge and experience with others in the field.

## Conclusion

Difficulty attending school jeopardises youths’ access to the benefits of school, and thus their development. The Knowing What Works project yielded signposts that support the development and delivery of interventions for severe or chronic SR. The signposts are based on the views of youths, parents, and professionals associated with 21 SR interventions delivered in education and mental health settings in the Netherlands, as well as the views of youths, parents, and professionals as presented in publications based on studies conducted in other countries. The 14 signposts point professionals to key areas warranting attention when addressing the multifaceted problem of SR. Ultimately, the signposts may improve the effectiveness of SR interventions, reducing absenteeism and dropout and thereby increasing adaptive development and positive futures for youths.

The proposed set of signposts serves additional functions. First, it relieves professionals of the time-consuming task of developing an intervention from scratch. Second, it serves as a checklist for professionals wishing to review and fine-tune an existing intervention or identify areas for team professional development. Third, it presents an organizing framework for the interpretation and assimilation of results from future studies and for developing new research questions. Fourth, it may be informative for policymakers as well as youths and parents seeking support.

The extent to which the signposts help professionals develop and deliver interventions for severe and chronic SR needs to be investigated. Moreover, the relevance of the signposts for other types of attendance problems (i.e., truancy, school withdrawal, school exclusion), for different school types (primary or secondary education, mainstream or special education), and for different forms of collaboration (monodisciplinary school model or multidisciplinary whole-school approach) are issues that deserve further consideration. In the meantime, the signposts offer a systematic approach to developing and delivering SR intervention for youths in different educational contexts.

Finally, the assumption is that school personnel are open to promote school attendance and prevent school absenteeism (Tier 1 of the MD-MTSS model), to respond efficiently when SR is emerging, mild, or moderate (Tier 2), and to collaborate with helping professionals inside and outside the school setting. This joint effort in Tier 1 and Tier 2 of the MD-MTSS model will help reduce the chance that youths experience severe and chronic SR.
